# Ultrasound for Breast Cancer Screening in Resource-Limited Settings: Current Practice and Future Directions

**DOI:** 10.3390/cancers15072112

**Published:** 2023-03-31

**Authors:** Qing Dan, Tingting Zheng, Li Liu, Desheng Sun, Yun Chen

**Affiliations:** Department of Ultrasound, Peking University Shenzhen Hospital, Shenzhen Peking University-The Hong Kong University of Science and Technology Medical Center, Shenzhen 518036, China; qingdan@pkuszh.com (Q.D.); kyzs_018@126.com (T.Z.); liuli@pkuszh.com (L.L.)

**Keywords:** breast cancer, screening, ultrasound, women’s health, low resource

## Abstract

**Simple Summary:**

Breast cancer (BC) screening is significantly important for reducing disease mortality. Mammography (MAM) is the gold standard for BC screening in high-income countries, while it is usually unavailable and infeasible in low- and middle-income countries (LMICs). Ultrasound (US) has been widely employed as an adjunct to MAM, particularly showing advantages over MAM for women of younger ages and with dense breasts. Nevertheless, it remains controversial whether US could be utilized as a primary tool for BC screening in underserved settings. This review focuses on randomized controlled trials and observational studies that demonstrated the role of US in BC screening. Furthermore, advanced techniques that might be useful to improve BC screening in LMICs are discussed. The results suggest that US, showing high sensitivity and an early detection rate, holds promise to achieve cost-effective screening initiatives where MAM is not available. The resource-appropriate recommendations on implementing BC screening in LMICs are also presented.

**Abstract:**

Breast cancer (BC) is the most prevalent cancer among women globally. Cancer screening can reduce mortality and improve women’s health. In developed countries, mammography (MAM) has been primarily utilized for population-based BC screening for several decades. However, it is usually unavailable in low-resource settings due to the lack of equipment, personnel, and time necessary to conduct and interpret the examinations. Ultrasound (US) with high detection sensitivity for women of younger ages and with dense breasts has become a supplement to MAM for breast examination. Some guidelines suggest using US as the primary screening tool in certain settings where MAM is unavailable and infeasible, but global recommendations have not yet reached a unanimous consensus. With the development of smart devices and artificial intelligence (AI) in medical imaging, clinical applications and preclinical studies have shown the potential of US combined with AI in BC screening. Nevertheless, there are few comprehensive reviews focused on the role of US in screening BC in underserved conditions, especially in technological, economical, and global perspectives. This work presents the benefits, limitations, advances, and future directions of BC screening with technology-assisted and resource-appropriate strategies, which may be helpful to implement screening initiatives in resource-limited countries.

## 1. Introduction

Female breast cancer (BC) is the world’s most prevalent cancer and remains the major cause of cancer-associated deaths globally. Based on the estimates from GLOBCAN 2020, there were about 2.3 million women diagnosed with breast cancer and 685,000 breast cancer-associated deaths worldwide [1]. BC has the highest incidence rates in high-income countries (HICs), whereas the BC deaths are highest in most low- and middle-income countries (LMICs) [2]. According to the Global Breast Cancer Initiative Implementation Framework from the World Health Organization (WHO), five-year survival rates for BC in HICs account for over 90%, compared with 66% in India and 40% in South Africa. Additionally, mortality rates of breast cancer in most HICs have decreased over time but remain high and increasing in many LMICs [3]. This disparity could be due to the late detection, inadequate diagnostic and treatment services, and low health coverage in LMICs [4].

It is well acknowledged that implementation of effective early detection programs is the first step to improve BC outcomes. Mammography (MAM) has been utilized as a gold-standard screening tool for BC in developed countries and has significantly decreased BC mortality, with a reduction of above 20% in women aged 50–69 and about 30% in women aged ≥ 70, respectively [5]. Nevertheless, it lacks meaningful benefits in women aged 40–49 and shows reduced accuracy in dense breasts, which not only could mask an underlying tumor on mammogram, but is also an independent risk factor of BC [6,7]. Furthermore, MAM is not readily available in under-resource settings because of the high cost and healthcare personnel shortage. It is reported that LMICs have less than 1 MAM unit per million people compared to 23 per million people in HICs [4]. This disparity, to a great extent, has contributed to the unfavorable BC detection in LMICs. Additionally, most cases of MAM screening projects run in LMICs have been evaluated as ineffective and unsustainable for a large population due to scarce resources [5,8,9,10,11].

Compared to MAM, ultrasound (US), including handheld ultrasound (HHUS) and automated breast ultrasound (ABUS), is low-cost, radiation-free, portable, and available. It is typically helpful for distinguishing between a cystic and a solid mass, which has been used as a second-look tool in women with mammographically occult lesions [12]. Emerging evidence demonstrates that US, compared to MAM, shows similar overall accuracy, increased sensitivity and detection rates, and relatively lower specificity [13,14,15,16].

There remains conflicting evidence whether US could be utilized as a primary tool rather than a supplement to MAM in BC screening initiatives in LMICs. Furthermore, the current reviews in this field have not comprehensively compared US and other main screening tools, highlighted novel techniques including artificial intelligence (AI) and portable screening devices that could empower US, nor presented resource-appropriate strategies for BC screening. Therefore, this review aims to summarize available evidence by analyzing the advantages and disadvantages of US in BC screening, discussing the clinical performance of US and the state-of-the-art techniques that might be helpful to increase the screening efficacy of US. Resource level-based recommendations for future BC screening in LMICs are also presented. This work will provide new insights for future research and practice in global women’s health.

## 2. Main BC Screening Tools

**Figure 1:**
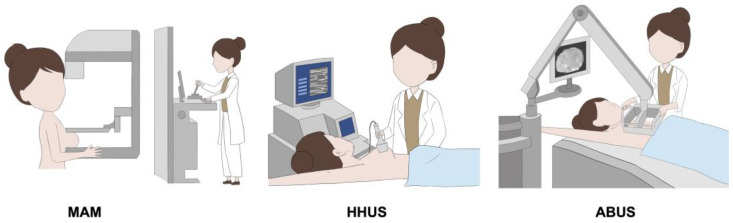
The schematic illustration of MAM, HHUS, and ABUS.

BC screening programs aim at the early detection of tumors in order to achieve the lowest morbidity for individuals and least medical cost to society. Here, we summarize the main screening tools, including MAM, HHUS, and ABUS, in terms of screening method (Figure 1), diagnostic performance, and economic cost.

### 2.1. MAM, HHUS, and ABUS

Currently, MAM is the only validated screening tool that can detect BC at an early and curable stage. The past decades have witnessed the significant achievement of MAM in reducing BC-related deaths and improving women’s health. According to the data from 2007 in the UK, among 1000 women aged 50 who underwent biennial MAM for 20 years, 2 to 3 BC-caused deaths were avoided [17]. Although MAM has been evaluated via several randomized controlled trials (RCTs) since 1980 and before its wide recommendation and implementation [18], different methods used in those trails led to the variable mortality reduction. Moreover, the main issue of MAM is the decreased sensitivity in dense breasts. BC usually occurs 10–15 years earlier in Asian women compared to women in western countries [19,20,21]. Asian women, particularly of younger ages, tend to have dense breasts [22,23], which makes it more difficult to distinguish between abnormal and normal breast tissues using MAM [24,25,26,27,28]. Additionally, there remain several intrinsic limitations of MAM, including few availabilities to LMICs and ionizing radiation hazards to examiners and patients. The benefits and harms of MAM, therefore, have been continuously debated. In this sense, HHUS and ABUS have been employed as adjunct tools to MAM for BC screening.

HHUS as a non-invasive, ionizing radiation-free imaging technique has been utilized for diagnosing breast disease since the 1970s [12]. It can delineate morphological characteristics and internal structures and accurately measure breast abnormalities. Particularly, US is useful to detect lesions in dense breast tissues, which are often difficult to visualize using MAM [15,29,30,31,32]. Furthermore, if additional tests are recommended, such as a biopsy, US is the ideal tool to guide subsequent procedures [33]. Additionally, The Royal College of Radiologists (UK) recommends US as the primary examination in symptomatic women aged 35–39 [12]. It is now generally acknowledged that US should be used as a first-line imaging modality in woman under 35 years and as a further assessing tool for palpable and mammographically detected abnormalities in all patients [12]. However, HHUS is operator-dependent, leading to poor reproducibility in diagnostic accuracy and examination time needed for image acquisition and interpretation.

ABUS is based on automated breast scanning with a 5–14 MHz linear array US transducer which generates three-dimensional breast tissue images [34,35]. It is designed to standardize breast US, increase reproducibility, and reduce operator-dependence and time for examinations and interpretations [36]. Basically, patients are in a supine position, then ABUS starts acquiring images after placing the probe over the breasts with only mild compression [37] (Figure 1). The image acquisition time is usually consistent in exam workflows, which can properly allocate time slots for every patient [38]. Images are then reconstructed in three dimensions for the radiologists to interpret in a separate workstation, which simplifies the screening workflow and reduces the whole examination time compared to HHUS. It is reported that the image acquisition time of ABUS is approximately 15 min per patient [39]. In contrast, imaging acquisition of bilateral breasts per patient using HHUS takes 19 min on average [40,41]. The interpretation time by radiologists ranges from 3 to 10 min, depending on differences in the complexity of each case and radiologists’ experience [42]. Of note, ABUS examination only requires technologists while HHUS requires qualified sonographers, or US physicians in some countries. However, ABUS shows an inability to evaluate the axillary region, vascularization, and tissue elasticity. Unlike HHUS, it is also impossible to conduct invasive procedures under ABUS guidance. Therefore, how to incorporate ABUS into BC screening workflows in the best way remains an issue that requires further investigations.

In brief, a comparison of the advantages and disadvantages of MAM, HHUS, and ABUS is presented in Table 1. It is essential to maintain an appropriate balance between the merits and limitations of each screening modality.

### 2.2. Diagnostic Performance Comparison between MAM and US

Over recent years, with improvements in US image quality, US screening has become more feasible and more desirable. Several systematic reviews conclude that adjunct US screening could detect suspicious breast lesions missed by MAM, with a higher detection rate and diagnostic sensitivity for women with dense breasts [13,14,16,43,44]. Some HICs, including Finland, Austria, Belgium, Monaco, and Italy, have evaluated the performance of US as a supplementary tool for population-based BC screening [45].

US shows a higher sensitivity and detection rate than MAM, particularly for women with dense breasts or of younger ages. Generally, supplementary US examination after negative MAM increased cancer detection (1.8–4.2 per 1000). A study in Italy [46] evaluated the performance of breast US in 22,131 asymptomatic women with negative tests in MAM. Incremental cancer detection rates in women aged <50 years (1.95 per 1000) and women with dense breasts (2.21 per 1000) were observed. Another RTC (J-START) in Japan [47] enrolling 72, 998 women showed that screening sensitivity of MAM + US (91.1%, 95% CI: 87.2–95.0%) was significantly higher than that of MAM alone (77.0%, 70.3–83.7%; *p* = 0.0004), with a remarkable reduction in specificity (87.7%, 87.3–88.0% vs. 91.4%, 91.1–91.7%; *p* < 0.0001). Additionally, the cancer detection rate was higher in MAM + US than that of MAM alone (0.50% vs. 0.32%, *p* = 0.0003). Particularly, the trial found that, for dense breasts, the sensitivity of MAM alone was 74% (95% CI: 69–79%), while MAM + US showed a significantly higher sensitivity of 96% (95% CI: 93–97%), indicating that US could detect some mammographically occult lesions. However, screening specificity in dense breasts remarkably decreased from 91.4% (95% CI: 91.1–91.7%) in the MAM alone group to 87.7% (95% CI: 87.3–88.0%) in the combined assessment group. Other similar studies [15,29,30,31,48] also showed that the overall sensitivity of MAM was 65–91%, while it could decrease to between 47.8–64.4% in women with dense breasts, leading to the omission of a certain proportion of malignancies. Moreover, MAM screening disparity in HICS and LMICs is reported. For example, in the United States, the MAM has an overall sensitivity of 87.8% whereas the sensitivity in LMICs could decrease to 63% [49,50], suggesting that MAM in LMICs is not as feasible as in HICs.

US detects small, invasive, node-negative, early-stage cancers (stage 0 or I) [48,51]. Boo-Kyung and coworkers compared the BC seen on a sonogram and mammogram. The mean size of the invasive tumor was 1.0 cm in the US-detected lesions and 1.6 cm in the MAM-detected groups (*p* < 0.001) [52]. According to recent reports [53,54], above 90% of women with stage I or II breast cancer will survive five years or longer, whereas the five-year survival rate greatly drops in BC above stage II. It is well acknowledged that early detection usually brings about higher survival rates and lower medical costs. US has been found to sensitively detect BC in early stage, such as stages 0, I, and IIA [47,51], which are usually associated with a good prognosis. Nevertheless, it is controversial whether the increased cancer detection rate of US could reduce BC mortality.

US decreases the interval cancer (IC) rate. The IC rate is given between two rounds of screening and is considered as an indicator of quality and efficacy in BC screening programs [55,56]. Dense breasts are a marker of increased risk of IC in screening. Compared to fatty breasts, extremely dense breasts show a 17.8-fold increase in the probability of IC [24,25,28]. In addition, these women with IC often present locally advanced and/or node-positive BC [26]. A study reported by Vittorio Corsetti and coworkers demonstrated that supplemental US could bring the IC rate in women with dense breasts down to a similar level of non-dense breast patients, suggesting that additional cancer detection via US was likely to improve screening benefits in dense breasts [57].

### 2.3. US as a Primary Tool in BC Screening

Since MAM is less effective in younger women as well as women with dense breasts, US as a primary screening tool has been put forward and implemented in some countries where MAM is not readily available. Here we respectively describe the studies in HICs and LMICs.

A prospective RTC (ACRIN 6666) conducted by the American College of Radiology Imaging evaluated the performance of US as the primary screening tool. It reported that the US yielded a comparable cancer detection percentage to MAM (52.3% vs. 53.2%, *p* = 0.9), with a higher proportion of invasive cancers and node-negative cancers. However, greater recall and biopsy rates as well as a lower positive predictive value of biopsy were more commonly seen in US than MAM [51].

In contrast, in low-resource settings, a recent meta-analysis with a total of 76,058 patients demonstrated that US had the potential to be an effective primary BC detection tool [16]. In six BC screening trials [51,58,59,60,61,62] from LMICs, including Argentina, China, Nigeria, and Malaysia, US showed a pooled sensitivity of 89.2% and specificity of 99.1%. Notably, women in LMICs often present with advanced stages and younger ages. In this context, they have a higher likelihood to benefit from US than MAM [63].

A multicenter RTC in China demonstrated that US could be used as a screening tool to detect BC in high-risk (e.g., dense breasts) women aged 30–65 years. It showed that US, compared to MAM, had higher sensitivity (100% vs. 57.1%, *p* = 0.04) and accuracy (99.9% vs. 76.6%, *p* = 0.01), comparable specificity (99.9% vs. 100%, *p* = 0.51), and lower screening cost, which was only 17.4% of MAM and 36.5% of MAM + US screenings [58]. Additionally, in the ‘Two Cancer Screening’ campaign in China, US was employed as the primary option for BC screening in 1.46 million women aged 35–59 years [21,64]. These findings suggests that, in developing countries, US could play a primary role in BC screening when MAM is not accessible and acceptable for women.

Afterwards, Li Yang et al. conducted a cost-effectiveness analysis of a BC screening program in China. It was found that compared with no screening, the screening program led to higher cost in rural China, with an incremental cost-effectiveness ratio (ICER) of $916 per quality-adjusted life-year (QALY). In contrast, for urban women who generally were at higher BC risk and more willing to pay for breast health management, the screening services cost $84.99 and gained QALYs of 0.01, with an ICER of $6671 per QALY. The authors concluded that rural women in China had low BC incidence, so general population-based screening for asymptomatic women at an average risk of BC was not cost-effective. However, compared to no screening, screening for high-risk women in urban China was very likely to be cost-effective [65]. An up-to-date BC guideline for China recommends US as the primary screening test for high-risk women aged between 40–44 years [66].

Although a few RTCs and evidenced-based systematic reviews evaluated the feasibility of US as a primary screening modality of BC, global recommendations have not yet reached a unanimous consensus due to the lack of evidence for reduced mortality with US screening. Because of the lack of good data management and research resources currently, further studies are needed to gain deeper insights into BC screening with US in LMICs.

### 2.4. ABUS in BC Screening

Kelly and coworkers conducted a multicenter study that screened 4419 women with MAM alone and MAM + ABUS. The participants were characterized with dense breasts and/or increased risk of BC. It was found that ABUS improved the detection rate from 3.6 per 1000 cases (MAM) to 7.2 per 1000 cases (MAM + ABUS). Sensitivity increased from 40% to 81% by adding ABUS. Additionally, the positive predictive values of biopsy were 39% for MAM and 38.4% for ABUS, respectively. Similar to HHUS, the recall rate of ABUS was also elevated, with 9.6% of MAM + ABUS and 4.2% in MAM alone [67]. Many studies compared the performance of ABUS and MAM in screening settings, showing improved sensitivity, detection rate, and recall rate in ABUS (Table 2). Of note, among these studies, cancers detected only by using ABUS were predominantly small-size, invasive, and node-negative [68,69,70,71]. When combining with MAM, ABUS plays an important role in screening programs to overcome the limitations of MAM.

To evaluate the diagnostic performance of ABUS as the primary screening method for BC, a multicenter prospective study in 2020 examined 959 asymptomatic Korean women aged between 40–49 years. The cancer detection of ABUS was 5.2 per 1000, higher than MAM (2.7 per 1000). ABUS also had favorable sensitivity, specificity, and accuracy ratings of 83.3%, 90.7%, and 90.6%, respectively. It suggested that ABUS could probably be an alternative to screening MAM among women aged between 40–49 years [75].

Current studies also compared the performance of ABUS and HHUS. As shown in Table 3, the performance of ABUS and HHUS was evaluated in 5566 women, with ABUS showing increased sensitivity and specificity [68]. Other studies in smaller populations also reported that ABUS had higher sensitivity than HHUS (92.5–95.3% vs. 88.1–93.2%) and comparable specificity (80.5–91.9% vs. 82.5–88.7%) [69,70,71]. However, other studies showed that ABUS had significantly lower sensitivity [76,77]. Overall, the variable diagnostic metrics probably resulted from the different study methods used in these studies. Future RTCs that separately compare MAM, HHUS, and ABUS for BC screening, particularly in LMICs, are necessary to conclude which is the better screening option. However, it could be time- and money-consuming to conduct these studies.

## 3. Novel Techniques in US for BC Screening

In the following section, we focus on some new techniques and feasible measures that would facilitate US in BC screening in LMICs.

### 3.1. Computer-Aided Detection (CAD) in ABUS

Due to the considerable amount of ABUS images, reviewing a full ABUS examination can be burdensome and malignant lesions may be overlooked. CAD software has been introduced to assist radiologists in interpretating images and generating accurate diagnosis [78,79,80,81], which would be a promising solution in LMICs with a lack of healthcare staff. A study in China evaluated the role of CAD in decreasing ABUS reading times and increasing the diagnostic accuracy of junior radiologists [82]. It demonstrated that CAD helped inexperienced readers to improve cancer detection accuracy in asymptomatic women. In the reading study, all radiologists could save 32% of the reading time among 18 radiologists by adding ABUS without compromising the diagnostic accuracy. Additionally, the mean sensitivity of less experienced radiologists increased from 67% to 88% by using CAD in the second-reading mode and concurrent-reading modes (*p* = 0.003). In this sense, several commercial CAD-ABUS systems (i.e., QVCAD, Qview Medical Inc., Los Altos, CA, USA) have been clinically applicable and tested for diagnostic accuracy and efficiency compared with radiologists [83,84]. CAD systems have promising potential to improve diagnostic accuracy and decrease the interpretation time of radiologists.

### 3.2. Deep Learning (DL) in ABUS

**Figure 2:**
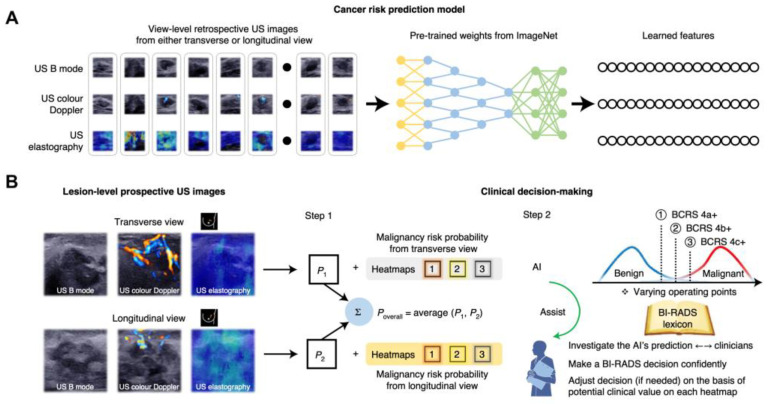
A DL-based system for BC risk prediction [93]. (**A**) The construction of DL model. The DL model was developed using multimodal US images (including US B-mode, US color Doppler, and US elastography), trained through multiple layers from ImageNet, and subsequently acquiring features. (**B**) Cancer prediction via DL model and clinical decision-making. The DL system inputs multimodal US images and outputs an overall probability of malignancy. According to the BIRADS lexicon, three different breast cancer risk scores (BCRS 4a+, BCRS 4b+, BCRS 4c+,) were proposed in the prediction system to assist radiologists to make clinical decisions.

DL is a branch of AI and has drawn great attention over the past years in breast imaging. DL algorithms pass image information through a convolutional neural network, which processes pixel information and passes that information onto subsequent layers for eventual image classification (Figure 2) [85]. So far, various DL models have been applied to BC screening workflows [86,87,88,89,90,91].

Various preclinical studies have found that, compared to radiologists, the diagnostic accuracy in BC could be improved with the assistance of DL models [84,92]. Hejduk et al. trained and tested a deep convolutional neural network using 645 ABUS datasets from 113 patients to classify breast lesions. In a comparison study between DL model and two radiologists, the DL model yielded an area under the curve (AUC) of 0.91 (95% CI: 0.77–1.00), comparable to radiologist 1 (AUC: 0.82 [95% CI: 0.68–1.00]) and radiologist 2 (AUC: 0.91 [95% CI: 0.77–1.00]). The DL model showed a similar sensitivity as well as a higher specificity, positive predictive value, and negative predictive value. These findings suggested that the developed DL model could detect and distinguish breast lesions in ABUS with similar accuracy as experienced radiologists. In China, a population-based BC screening with DL-assisted ABUS is underway. It aims to have three million women screened for BC by 2023 via DL-based ABUS alone in asymptomatic women in rural China.

### 3.3. Portable US Devices in LMICs

In low-resource conditions, the poor facilities and unstable power grid make it difficult to install and employ high- or middle-end US machines. Smartphone/tablet-sized, battery-powered US devices hold great promise to satisfy the demands in underserved nations since they are portable, low-cost, and can be modified according to customized applications. For example, Ghana explored the use of portable US devices in community healthcare facilities for obstetric, pelvis, breast, vessel, abdomen, and genitourinary system examinations [94]. China also reported the construction of a portable US-assisted BC screening system [95]. More inspiringly, Mexico conducted a pilot study that built a DL model and incorporated the model into a low-cost portable US machine to triage the breast lesions. In this study, three healthcare staff without ultrasound experience were recruited to use the portable US system to acquire breast US images from 32 patients, then these images were analyzed using a previously trained DL model. Results demonstrated that the US device could be easily operated by these healthcare workers and the built-in DL model had a similar diagnostic accurate as breast radiologists [96]. It provided a new strategy of implementing cost-effective BC screening services in scarce-resource settings with a lack of equipment and healthcare specialists. In the future, population-based RTCs should be conducted to validate the possibility of utilizing AI-enabled portable US systems for BC screening.

## 4. Implementation of US for BC Screening in LMICs

As discussed above, US (i.e., HHUS and ABUS) shows some unique advantages over MAM, especially in LMICs, such as sensitivity to dense breasts, low cost, acceptance by patients, and wide availability. However, US is also imperfect. It shows decreased specificity, and HHUS requires experienced sonographers to perform a handheld exam. There is still insufficient evidence recommending the utility of US as a primary screening tool in LMICs. However, in certain settings, US is helpful for improving women’s breast health. According to the Global Summit Early Detection Panel and the BHGI, screening initiatives could be implemented based on national health resources (basic, limited, enhanced, and maximal) [63]. It suggested that in limited-resource settings, combining clinical breast examinations and breast US may be an acceptable approach.

For BC screening programs, a key question is to what extent mortality is reduced in relation to results from screening services, since the observed mortality reduction could be attributed to other dominants such as improved awareness and management of BC. The benefits of screening should not be inappropriately propagated without addressing the harms, such as false positives and overdiagnosis. False-positive recall, which increases the number of unnecessary recalls for further interventional tests, is deemed as one of the main barriers to implement BC screening programs. Overdiagnosis, where women are diagnosed with BC which are proven to be non-life-threatening during their whole life [9], causes unnecessary psychologic stress and is a waste of resources in the following treatments [9,97]. Due to the high sensitivity, false-positive recalls and overdiagnosis of US cannot be overlooked. With improved experience and revised interpretive criteria, the false positives of US can be reduced.

Overall, it is essential to weigh the benefits and risks in every screening program. In this sense, we present the recommendations for BC screening with US, which could be helpful to improve the effectiveness of employing US in BC screening in LMICs.

### 4.1. Data Management

Accurate data, such as incidence, mortality, and survival data, are crucial for BC screening guideline proposals and screening resource allocation. While the data are often found missed or poorly managed in LMICs [21,64], establishing regional population-based cancer registries and data documentation are recommended.

### 4.2. Public Awareness

The lack of public awareness of breast health is a great barrier to BC screening programs. Raising BC awareness and establishing a breast health culture are cost-effective control strategies. It probably could be achieved by involving various interventions, including community-based education in rural areas and creating partnerships with religious communities [98]. Besides the screening benefits, all potential participants should also be clearly informed about the potential harms.

### 4.3. Target Group

Since it is not possible to screen all women, including the low-risk potential participants in LMICs, the cost-effective approach is to target elevated-risk populations based on age, breast density, genetic mutations, family history, or other personal risk factors.

### 4.4. Effective Treatment

Compared to any screening program alone, it is more likely to decrease BC mortality by developing adequate treatment facilities where patients are able to receive timely and effective treatments. Easy accessibility to and greater affordability of cancer care facilities are crucial for the successful implementation of any BC programs. Otherwise, screening services would be a pure waste of resources.

### 4.5. Novel Techniques

Apart from its advantages, US has intrinsic limitations for BC screening. Hopefully, these flaws could, to some extent, be compensated with the development of novel technology (i.e., smart portable devices, DL detection/classification systems). These techniques, expected to be feasible solutions to the lack of healthcare staff and screening machines in LMICs, should be investigated further and incorporated into the workflows in real-world BC screening programs.

## 5. Conclusions

BC screening is an essential step in decreasing the global burden of BC. Although MAM is a gold-standard screening tool in HICs, it is not always available in LMICs, and it is not recommended for younger women or women with dense breasts. US (including HHUS and ABUS), showing many advantages over MAM, may be suitable in certain settings where MAM is unavailable or unfeasible. When enabled with novel techniques, such as DL and smart portable devices, US holds great promise for BC detection, while further trials are needed to validate the utility of US as a primary BC screening tool in LMICs. To achieve high cost-effectiveness and optimize benefits to potential screened participants, multiple factors, such as local resources, risk factors, and religious and cultural values, should be comprehensively considered before implementing BC screening services.

## Figures and Tables

**Table 1:** Comparison of MAM, HHUS, and ABUS for BC screening.

	MAM	HHUS	ABUS
Sensitivity to dense breast	Low	High	High
Sensitivity to microcalcification	High	Low	Low
Specificity	High	Decreased	Decreased
Reproducibility	High	Low	High
Guiding further biopsy	Non	Yes	Non
Radiation	Yes	Non	Non
Breast compression pain	Yes	Non	Non
Equipment availability	Less	Wide	Less
Examination expense	Relatively expensive	Less expensive	Less expensive
Examination provider	Technologist	Experienced sonographer	Technologist

**Table 2:** Comparison of diagnostic metrics of MAM and MAM screening plus ABUS. Abbreviation: NR, not reported.

References	Patients	Sensitivity (%)		Specificity (%)		Detection Rate (per 1000 Women)		Recall Rate (per 1000 Women)	
		MAM	MAM + ABUS	MAM	MAM + ABUS	MAM	MAM + ABUS	MAM	MAM + ABUS
Giuliano [72]	3418	76	96.7	98.2	99.7	4.6	12.3	NR	NR
Brem [39]	15,318	73.2	100	85.4	72	5.4	7.3	150.2	284.9
Giger [73]	185	57.5	74.1	78.1	76.2	NR	NR	NR	NR
Kelly [67]	4419	40	81	95.15	98.7	3.6	7.2	42	96
Wilczek [74]	1668	63.6	100	99	98.4	4.2	6.6	13.8	22.8

**Table 3:** Comparison of diagnostic metrics of ABUS and HHUS.

References	Patients	Sensitivity (%)		Specificity (%)	
		ABUS	HHUS	ABUS	HHUS
Choi [68]	5566	77.78	62.5	97.8	96.7
Wang [69]	213	95.3	90.6	80.5	82.5
Wang [70]	155	96.1	93.2	91.9	88.7
Chen [71]	175	92.5	88.1	86.2	87.5
Niu [76]	173	40	92.23	77.62	80.24
Jeh [77]	173	88.05	95.7	76.25	49.4

## Data Availability

Data supporting reported results beyond what is reported in this manuscript are available upon reasonable request from the corresponding authors.
